# Introductory Lectures

**Published:** 1857-01

**Authors:** 


### INTRODUCTORY LECTURES.

We are in receipt of several introductory addresses delivered at the opening of the present term of Winter Lectures on Medicine; and first, we have the "Lecture Introductory to the Course of Institutes and Practice of Medicine, in the Medical College of the State of South Carolina, by Samuel Henry

Dickson, M. D., LL. D., Charleston, November 3d, 1856." Of this we can say, as of everything from the pen of the distinguished author, that it is very interesting.

In considering the elements which go to constitute the character of the true Physician, he remarks:

"If you estimate this character at its proper value, if you regard it with the respect and veneration to which it is indeed entitled, you will resolve to spare no pains which shall be necessary to make it your own. Let us enumerate and dwell briefly upon a few of the more prominent of these elements.

"The Physician must, above all other men, be *diligent.* Think how much is to be done, and recollect that we have but one short life to spend upon it. We engrave for eternity; let no day pass without its added line: '*nulla diee sine linea;*' we have no time to lose. To raise a proper superstructure, the foundations must be laid deep and wide. Each step must lead on to another; nothing is infertile; nothing transient; whatever is done, remains; we paint in fresco, the picture and the ground on which we lay our colors, hardening into stone as we paint. I do not know any individual more entitled to pity than a high minded and conscientious young man, who, upon entering the ranks of our profession, finds that his early education has been defective, and that he labors under the heavy disadvantage which such deficiency must necessarily entail upon his whole life career. He will not be consoled, even by the thought that he has not been alone, or chiefly, who is to blame for this great error; that he is partly the victim of a bad system, and partly of an unjustifiable impatience peculiar or almost peculiar to our country, which urges to enterprise and action the immature adolescent before his faculties have reached their due development, and thus denies him the reasonable opportunity for requisite preparation and preliminary studies. He will find abundant consolation, however, in the reflection that it is never too late to begin a correct course—and that 'nothing is denied a well-directed labor, though nothing is attainable without it,' and in the fixed resolve carried out without delay, and with steady perseverance, that he will comply with the requisitions of the contingency, and raise himself step by step, to the lofty height he aspires to reach.

"'Take comfort, my son,' wrote the illustrious Chatham to the no less illustrious Boy Minister, who so long governed the mighty Kingdom of Great Britain, 'Take comfort, my son! after all, you have only the Cyclopedia to master.' The domain of Scientific Medicine reaches to-day over the vast realms of modern attainment, comprising within its recognized limits an acquaintance with every department of human knowledge. 'What science is there'—exclaims a recent writer on Physiology, himself an admirable example of omnivorous acquisition —'What science is there which is not involved in explaining our structure and functions? Anatomy, Chemistry, Zoology, the various branches of Natural Philosophy, which themselves require as their foundation Mathematics,' are briefly specified, but it would be difficult to say what could be excepted.

"Little less, indeed, than the complete circle so tersely indicated by Chatham, will suffice for the Physician. 'To learn to reason, he must know.' Knowledge is conveyed and bound up in language. Classical learning, therefore, and what is called literature, are necessary for him as truly as science proper, that he may understand what he reads, and be capable to state, to narrate, to infer, to argue, to prove, to convince. Modern languages are indispensable to him, if he aim at the highest point of excellence and its prompt attainment. It is mortifying to be compelled to wait for translations; and still more, to depend upon others not only for the selection of what they choose to distil for us from foreign materials, but for the correctness and truthfulness of the conveyance. I am aware that 'all is not possible to all,' nay, perhaps not to any; but I am anxious to impress upon you the urgent demand that you should attain to all that *is* possible, each one for himself. It is infinitely easy to know too little; infinitely dificult to know too much! Besides, each one has his peculiar or special aptitudes, which he will never discover, nay, which have no means of developement, but through diligence, patient study, vehement mental effort. As in a course of gymnastics, we find ourselves, upon earnest and repeated trial, capable of feats of strength and agility unthought of and unhoped for, so I will venture to assure you, that the steepest and loftiest heights of the apparently impossible are often—and sometimes with unexpected ease too—scaled by the resolute climber. Forward, then, and be your motto, Excelsior! If your preliminary education have been imperfect, take up again, with manly determination, the grammar and the dictionary. Master your own glorious tongue, the language of Milton and Shakspeare and Bacon—of Sydenham and Cullen and Rush. Enable yourselves to communicate your thoughts in the fixed and stereotyped—not dead—but emphatically living and immortal dialect of universal science employed by Cicero and Pliny and Celsus, Stahl and Gregory. Open with familiar key the new and daily multiplying treasures accumulated by our French and German brethren, and it is impossible for me to exagerate the advantages and facilities which will accrue to you from these highly available sources of intelligence.

"The Physiology of the present day—which the sagacious Draper has labored to place among the positive sciences—its Pathology, its Diagnosis, its Therapeutics, are peremptory and exacting. The immense resources of Chemistry must be brought within your reach—its universally applicable principles, and at least so much of its minute details as belong to the relations of organized substances to each other and to the agents that surround them. Here there is nothing constant but change. Progress is perpetual. The truth of to-day is proved to-morrow to be error; yet it is fruitful error leading on to further and perhaps more fertile error, and still further at last to truth. Encircled and sometimes narrowly circumscribed by the *quid ignotum* all around and about us, the small area of uncertain light almost buried in the all-enveloping darkness, we feel our way from point to point, ever hopeful and undismayed, because ever and anon sure to be gratified by some new acquisition—some new foothold from which again we start on our ennobling career of unceasing advancement. Of all conquests possible to man, this triumph over ignorance is doubtless the most elevating."

We have also another Introductory from the same Institution, delivered by Prof. James Moultrie, at the commencement of the Course on Physiology, in reference to which noble science, he remarks:

"It is apparent that physiology, as I have said, is once more reclaiming to itself its ancient extent and significancy; and to know it as it should be known, those who would enter upon the career which it prescribes, must fit themselves for it by varied as well as extensive acquirement. The physiology of to-day is vastly different from what it was in days of yore. Indeed, so greatly do they differ, that were it not for the historic links which unite them, it would not be possible to trace their genetical relationship. My occupancy of a Chair in this Institution, which has been exclusively appropriated to it, and the lectures which I yearly deliver, jointly with my several colleagues, evidences the estimation in which it is held, and its multifarious relations to what they teach, as well as to the essential attainments of a medical education. Other evidences of it, also, will necessarily and repeatedly be brought to notice in the course of the observations and reflections which it will be pertinent to make, in reference to comparative and human anatomy and physiology, and their kindred inquiries, psychology, and zoology. We shall have frequent occasion, too, to allude to the varied, general relations and conditions to which, as we have said, it is indebted for its origin and progress.

What ideas may prospectively be formed of its development and availability, we are in no condition now adequately to determine. From what has recently been accomplished, however, we are entitled to expect infinitely more. The services it has already rendered to psychology, fully justifies the remark. All the sciences have their being in the mind. Hence, psychology has been called the "Science of Sciences," and that all look to it for "first principles," to guide them in the several tasks which they have to perform. A clear and comprehensive natural philosophy of the faculties and operations of the understanding, has long been a desideratum in mental science: for the moralist turns to it for light to illumine the chambers of conscience; the legislator for a standard to measure the degrees of crime or of guilt, and a sanction to award their punishment; the educationist for data to organize systems of instruction, which shall conduce towards the promotion of, rather than counteract, the fundamental teachings of nature; the physician for correct and clear ideas of the nature of insanity, and apt contrivances for its prevention and remedy; and the theologian for a solution of the problem of the origin of evil, and a true elimination of those super-sensualities of the soul, which have been commissioned to lift it above the sphere of earth, and to wing it to those elevated regions, where it may commune with thoughts pertaining immediately with the Infinite. Their appeals, however, have not as yet been answered. While, in cotemporaneous language, it has borrowed light from all, it has shed none upon them in return. The question naturally arises, therefore, as to why is this? It is because psychology has been without an appropriate guide, and has for ages been wandering in speculation and hypothesis. Until very recently, its home has been in the region of the abstract; but it has, at length, passed from this, into the domain of the concrete. It has become a factor of physiology, and in common with it, commences to rebuild a new structure on the anatomy and function of the nervous system. For this change it is indebted to positive philosophy; and in its renewed activity, promises, from its recent gains, inconceivable advantages to be hereafter won in fields of inquiry that have only been partially explored.

"Who, in this new connection, have been most distinguished for their researches and successes, the annals of the present day very unequivocally declare. Among them, may be found the names of physiologists and physicians. And the reason of this lies in the special nature of their calling. The physician, unless he prove unworthy of the title which he bears, must necessarily be a physiologist. To him, for a long while, was entrusted the cultivation of this science and its congeners, and the exclusive management of the insane: an intelligent consciousness of the essential characteristics of that disorder, must always have been experienced by him, therefore, as a painful and pressing necessity. Out of this source consequently, has come much of the illumination which has been shed, and to which we owe better ideas of the nature of intelligence. The course of investigation into which he was thus led, and which he has pursued with so much diligence, relative to the structure of the brain, and spinal marrow, and nerves, together with their functions and disorders, as well as other related objects, could not fail to introduce new results, and thereby to prepare the way for, as well as to lay the foundations of, a renovated psychology, which, under the guidance of its new and positive methods of inquiry, bids fair to clothe itself, in due season, with the certitudes which have already invested its predecessors and cotemporaries.

"In elucidation of the above, it would be easy to cite examples; but passing by the names of many, it is sufficient to enumerate, from among the living, the conspicuous ones of Bell, of Hall, of Carpenter, and of Noble. With their labors, we shall become partly acquainted shortly. It is only proper to remark here, that these writers, in the important discoveries which they have made, relative to reflex action, instinct, intelligence and volition, have created an epoch in the history of that science. The nervous system, as an entirety, now stands forth, as the great organic mechanism of all their varied phenomena; the link between the forces within, and the forces which environ them without; the substratum, through which they severally correlate; and by means of which, the relations of the interior are enabled to co-ordinate with the relations of the exterior. Progress and development thus have substantial, as well as a speculative, basis, and its records are rendered permanent, as well as practicable and consecutive. Experience, the primordial basis of all knowledge, is provided with a fixed habitation, as well as a name; is no longer an abstraction, intrinsically and isolatedly related to mind; but a concrete, which has a being in staties as well as dynamics, in structure as well as action, in matter as well as force. Experience is organizable, and organized. It becomes, in other words, a constituent of organization, and may be integrated; and in this fact, lies the perpetuity of the sciences, and their improvement, as well as the perpetuity of society, and its civilization. The "organization of societies" a phrase so rife in human parlance, and so essential to the language of the statesman, the politician and the common people, agreeably to this doctrine, is not merely a metaphor, or fiction of expression, but a conventionalism, denotive of a structural and functional truth, having the same organic establishment in man, *ceteris paribus*, as that which the instincts have in the beaver, the bee, the termite and the ant. The essence of materiality, and the essence of immateriality, are propositions of the inconceivable, and no longer find entertainment within the legitimate exercises of philosophy. But if experience, which is science, and civilization, and thought, are concretes; if every thought is accompanied with some movement among the constituent primordials of the brain, and these give rise in turn to correspondent activities of thought; then may they severally and reciprocally entail upon one another the influences of their mutations, whether these be for good or for evil. This observation, let it be remembered, is momentous; for it affects alike the physical, and moral, and intellectual, and theological education of the world. If pedestrianism augments the muscularity of the leg, and the muscularity of the leg augments the capabilities of the pedestrianism; if labor at the anvil develops the muscularity of the arm, and the muscularity of the arm enhances the capabilities of the anvil; if thoughts alter the volume, too, and the form of the several parts of the brain, and those altered parts of the brain enlarge, by reciprocation, the comprehension, and adhesiveness, and versatility of thought; tnen have we attained a concrete explanation ot the fact, that many actions which, in their incipiency, are conscious and difficult of performance, by use are in due season rendered unconscious and easy; that the voluntary simulate the reflex, and the reflex the voluntary; and that those which, in the inferior races, are instinctive and automatic, in the superior, are changed into the intelligent and free. The law of unity of composition and of progressive development, thus finds its illustration and exemplification in the differentiations of species.

"Human society, for these reasons, as well as human civilization, is dependent, alike with comparative society, upon structure and function. Both have their seat in the neurine material of which that structure is composed, and in those correspondences of internal and external relations and adaptations, in which we have found the very essence of the experience which distinguishes the former, to consist. Hence its indelible registration, and transmissibility from generation to generation.

"It is the province only of superior minds, however, to contemplate the exalted relations which physiology has been thus imperfectly shown to bear to the hierarchy of science, or to carry forward the investigations to which their meditation leads. The subject has been introduced at this time, therefore, for the simple purpose of elucidating its variety and extent, and of affording a glimpse merely of that noble field, in which you are invited to labor, and to win, if you choose, an enduring crown. As it now presents itself to our reflections and study, it is the complex result of thousands of minds, professional and unprofessional, severally and unitedly at work, simultaneously and consecutively, century after century; and in all that lengthened and lengthening period, there is no science, which has not, as has justly been remarked, been tributary to its progress. Who, then, it may be asked, in view of this complexity and comprehensiveness, of its retrospective, present and prospective relations and developments, is competent to embrace it, or to undertake the instructions called for, by the requirements even of this Chair? I know of none. From its complete peformance, I do not hesitate to say, I involuntarily shrink. The fulfilment implies an amplitude of attainment, correctness of knowledge, and scientific readiness, which few are permitted to aspire to, and fewer still to attain.

"To the Medical Profession, in view of its own conceived utility and dignity, and as that dignity and utility are offered to the world, its cultivation and diffusion is of the utmost importance. It should not only be cultivated by its votaries, but it should be insisted upon as a separate and indispensable branch in every College, of a medical education; and no pains should be spared to introduce the study of its outlines into our elementary schools; so that children may acquire some knowledge of its value, and its extension thus become universal. No greater boon could be added to the provided means of civilization than this. It is the only specific against quackery. Professor Draper remarks that "Empiricism could not flourish as it does, if the structure and functions of the body of man were better understood.

"I rejoice at the efforts, therefore, which have recently been made to give it this direction; and that the success which has attended them, justifies their continuance. I rejoice, too, at the necessity which the freer diffusion of the knowledge of it will impose on candidates who offer for the honors and emoluments of the Diploma, to become well acquainted with the treasures which it contains. "A great revolution," I am persuaded, in common with the author before mentioned, "is impending in the practice of medicine," and that "in the future, the greatest physicians will be the greatest physiologists;" and in closing, take occasion to superadd my further testimony to his, that "He can best correct the imperfections of a machine, who best understands its structure and action."

In addition, we acknowledge the reception of the regular Introductory Address, delivered before the St. Louis Medical College, by M. M. Pallen, M. D., Professor, &c., which we can also cheerfully and warmly commend. In speaking of the wonderful developments of Modern Science and particularly of the telescope, the instrument of Astronomical Science, he continues:

"But it is not the instrument of the Physician. Yet there is one which belongs to him, whose revelations are no less wonderful: I allude to the microscope. Second only to the telescope, though in many respects superior to it, it transcends all others in its scientific value. It peers into the invisible life around us, and brings into view worlds in every direction—all space, within us, and without us, and around us, swarms with countless hosts. The zephyr, as it floats through the trees, fans in every leaf its millions of inhabitants. The air, which we respire, is replete with its myriads of aërial tiny travelers. The flints, the limestones, the tripolis, the soils, when examined with the microscope, proclaim the existence of untold numbers of living creatures in ages gone by; nay, more, they show that the very rocks we tread on are made up of them. Ehrenberg found that tripoli, a certain kind of silicious stone, is entirely composed of the remains of organic beings, there being about 41,000 millions of individuals in every cubic inch, or about 187 millions in a single grain. The city of Richmond, in Virginia, rests on a foundation composed of similar organic beings: the stratum, several feet in depth, extends a distance of twenty miles in length. It is curious to know, that Naturalists are not yet agreed whether these minute fossils, when living, belonged to the animal or vegetable kingdom, so indistinct is the line which divides the one from the other. The chalk cliffs of England are made of minute shells and fragments of shells.

'The dust we tread upon was once alive.'

'One of the greatest triumphs of the microscope has been to establish the unity of plan which prevails in organized structure. It is easy enough to say that the giant palm belongs to the vegetable kingdom, whilst the huge elephant which browses at its roots is an animal; but as we descend the scale of the animal kingdom and at the same time examine vegetation of a complex organization, it is dificult to define the limits of the one or the other. In the earlier periods of Zoölogical Science, spontaneous motion and the presence of a stomach were considered as the distinguishing characteristics of an animal; but we now know that certain plants (*confervæ*) possess the power of motion, and certain animals (sponges and allied families) are destitute of both. There was a time when nitrogen was assumed to be a distinct proof of the animal tissues, but it is now ascertained that nitrogenized matter necessarily belongs to the growth of the plants. The microscope, which was appealed to, to establish the differential line, proved that there is no distinctive character, and that all organized matter has a common origin. Who would have imagined that man has a common origin with the worm he treads upon! Who would have dreamt that the lordly lion, the crawling reptile, the majestic oak, the humble snowdrop, are all alike in the first elements! But it is true, that all animals and all plants spring from a primordial cell, which, in the vegetable kingdom continues to preserve its characters, and in the animal kingdom, undergoes a development into tissues —nerves, blood vessels, bone and muscle. A single cell is the first step in created life, and from the congeries of cells are developed those various parts of the noble casket which constitute man, and enclose his immortal spirit.

"In former times, the Anatomist was content to describe the position and relation of the organs of the body; but now the microscope penetrates into their hidden mysteries, reveals their minute structure, and reduces them to elementary tissues. It creates the science of Histology. Under its examination, the Past and the Present rise up as if by the enchanter's wand. Dig up from the rocks the tooth of a Preadamite animal, place a fragment under the lens, and the Naturalist reconstructs it, and gives you a picture of it as it existed in the days when there was no man to declare the glory of the Deity. Rivaling the fabled trial by bier-right, when the corpse of the victim was exhumed from its resting-place to confront the murderer, at whose dread touch its pure winding-sheet was reddened by the blood which poured forth from the wound as it opened its gory mouth to convict the accused, the microscope declares that his stained garment is polluted with the blood of his fellow-man; and even when time has drawn a curtain over the dark deed, the microscope and test-tube detect the secret poison and unveils the crime.

"To the Pathologist, the microscope is of immense value. It detects the lithic acid, the oxalates, the phosphates, the fungi, pus corpuscles, blood corpuscles, tubercular matter, and minute entozoa. It will even display a flower-garden in the stomach; but it cannot show a cancer cell, for the simple reason that none exist, as my colleague, Dr. Linton, reasoned out years ago, and as has been clearly shown by Rokitansky and others since.

"Wonderful are these revelations of the microscope! It teaches us that there is a hidden beauty in Nature all around us; it unrolls the scrolls of Time, and we read of the operations of Nature in ages gone by. But there are those who may ask, what is the use of such facts? They often ask, what is the use of any isolated facts in science? It is said when such a question was propounded to Benjamin Franklin, he replied by asking 'what is the use of a new-born baby?' No one could see the use of the infant philosopher when he was keeping his mother awake all night with the ills that babies are heirs to. Who would then have predicted his splendid discovery of the identity of lightning and electricity —his beautiful explanation of the action of the Leyden jar— his gift to mankind of the lightning rod? to say nothing of his aid to the great cause of American independence."

